# Dr Clive Noble – A Tribute

**DOI:** 10.17159/2078-516X/2022/v34i1a13095

**Published:** 2022-01-01

**Authors:** Jon Patricios

**Affiliations:** Professor of Sport and Exercise Medicine, Wits Sport and Health, School of Therapeutic Science, Faculty of Health Science, University of Witwatersrand

On the 14 January 2022, South Africa lost one its great sports medicine pioneers and medical characters. Dr Clive Noble was born in Johannesburg in 1938. In his youth he was more of an avid sportsman than an academic. He was a keen rugby player, school shot-put and discus champion, black belt in judo and boxed for the Germiston junior team. His academic progress was less stellar, somewhat frustrating his nursing sister mother’s ambitions of young Clive becoming a medical doctor. His 4 D’s, an E and an FF in Matric were, unsurprisingly, not adequate for acceptance into Wits Medical School!

## A determination to succeed

Determination prevailed however, and Clive was eventually accepted to Wits and had found his niche! He eventually graduated third in his class and was invited to become the Professorial Houseman, a great honour then. All the while, Clive continued his participation in sport. At Wits he took up weightlifting and became South African Universities’ and Southern Transvaal champion. This keen interest in sport and personal experience of the body’s physical capabilities developed a curiosity in injuries associated with sport.

## A growing interest in sports injuries

Clive decided that the best fit for him was to specialise in orthopaedic surgery. Sports medicine was decades away from becoming a recognised field. Clive was meticulous in documenting the results of his operations. The outcomes would be written up and presented in the talks he became renowned for in South Africa and abroad. Soon after starting his orthopaedic practice in Johannesburg, Clive Noble’s reputation as “the sports injury expert’ was well established and he became the “go-to” doctor for any matter associated with sport and exercise. In fact, anyone who had any injury often sought Dr Noble’s expertise including rock star Rod Stewart! His ideas were later formalised into the “Pfizer Manual of Sports Injuries”, one of the first sports injuries books.

## Sport teams

His contribution to supporting South African sport was immense. He consulted to the Boxing Board of Control from 1963 – 1988. He chaired the Transvaal Rugby Medical Committee, served on the South African Rugby Medical Committee and supported touring teams including the British Lions and the All Blacks. Kaizer Chiefs and Mamelodi Sundowns were two of the football teams that made use if his expertise. In 1992 Clive was the medical officer for the South African Olympic Team on our readmission to international sport. During this era, if there was any question regarding an injured sportsperson, Dr Clive Noble would be the first person to call.

## Knowledge translation

Clive was a great speaker. Long before the era of PowerPoint, he would scribble a few notes on paper and hold audiences here and abroad keenly attentive as he shared his experiences and theories related to the management of sports injuries. Clive was invited to speak across the world where he carried the South African flag high speaking at orthopaedic and sports medicine meetings.[Fig f1-2078-516x-34-v34i1a13095]

**Figure f1-2078-516x-34-v34i1a13095:**
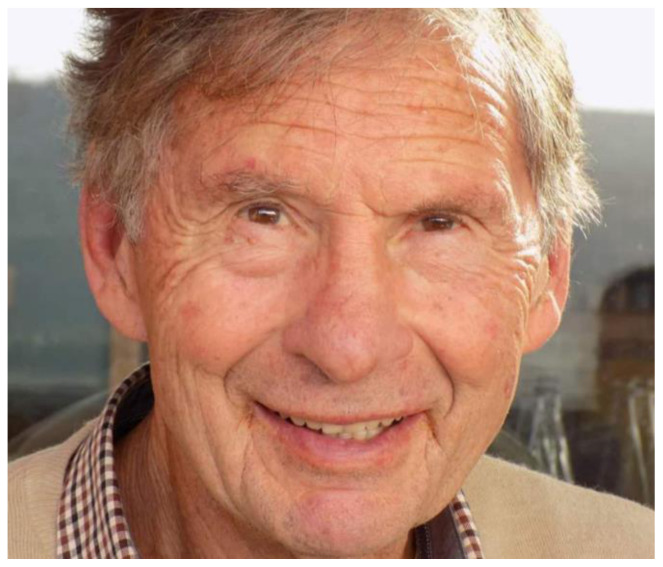


## SASMA and SAJSM are born

Clive, together with a few orthopaedic colleagues, recognised the need to develop a more formal sports medicine structure. In 1985, together with fellow orthopaedist Dr Ponky Firer, radiologist Dr Louis Sirken and a young sports scientist called Tim Noakes, they formed the South African Sports Medicine Association (SASMA) and Clive was voted the first President. SASMA hosted its first congress which was very well attended. The international network he had established allowed SASMA to invite prominent international speakers such as British orthopaedic surgeon Dr John Williams, Dr Wolfgang Pfoerringer, President of the German Society for Orthopaedic Sports Medicine and Paediatric orthopaedist Dr Lyle Micheli from Boston. Simultaneously Clive became the first editor of the South African Journal of Sports Medicine, a position he held for 10 years.

## South Africa’s first private sports medicine clinic

Clive’s travels made him aware of the need for multidisciplinary centres in South Africa that housed the expertise for managing sports injuries. For many years he had a dream of such a centre in South Africa and this was realised in 1995 when he opened the Centre for Sports Medicine in Johannesburg with fellow orthopaedists, a sports medicine physician, radiologist, physiotherapists, biokineticist and dietician. This model became the template for similar centres countrywide.

## An incredible human being

Despite having such an aura about him, Clive Noble had an ability to translate his profound knowledge so that it became easily understandable to junior clinicians as well as his patients. As Rudyard Kipling wrote, Clive could certainly “walk with kings but not lose the common touch.” A challenging patient would benefit from Dr Noble popping his head into a colleague’s consultation room for some sage advice, theatre time was spent learning (and sharing plenty of gags!) and after-hours he was always willing to provide telephonic advice. Clive was a great character. As much as he was serious in his approach to his patients, he also could appreciate a practical joke. He was a great raconteur and would regale audiences as he wittily reminisced about his experiences in medicine and life. He remained fit throughout his working life. Clive competed the Comrades Marathon and legend has it that his training consisted only of regular calf raises! At the Centre in Rosebank he would show up younger colleagues and the sportsmen being treated as he marched into the gym, rolled up his sleeves, set the chest press machine at maximum and performed a few repetitions.

## A legacy

In recognition of his pioneering contribution to South African sports medicine, the opening keynote address at the biennial SASMA Congress is fittingly named “the Noble Lecture”. But, for those of us lucky to have interacted, worked with and be trained by Clive Noble, his greatest legacy will be the transfer of incredible knowledge and skills from a generous, gregarious and humble human being with a passion for sports medicine. He shared with us a full and incredible life.

